# An Exceptionally Rare Ovarian Lymphangioma Mimicking a Dermoid Cyst Recurrence: A Case Report

**DOI:** 10.7759/cureus.94985

**Published:** 2025-10-20

**Authors:** Mohamed Hemdan, Rohit Arora, Weihong Ma

**Affiliations:** 1 Obstetrics and Gynaecology, Manchester University NHS Foundation Trust, Manchester, GBR; 2 Gynaecology, Manchester University NHS Foundation Trust, Manchester, GBR; 3 Histopathology, Manchester University NHS Foundation Trust, Manchester, GBR

**Keywords:** adnexal mass, dermoid cyst mimic, ovarian lymphangioma, rare benign ovarian tumour, recurrent ovarian cyst

## Abstract

Ovarian lymphangioma is an exceptionally rare benign tumour derived from lymphatic vessels, with only a few cases reported worldwide. The imaging features are nonspecific and often resemble those of common adnexal lesions, such as dermoid cysts, cystadenomas, or endometriomas. Therefore, a definitive preoperative diagnosis is difficult, making histopathological confirmation essential. Surgical excision, either by cystectomy or oophorectomy, is the treatment of choice, with a favourable prognosis when excised completely. However, because of the rarity of this condition, there are no established evidence-based recommendations for postoperative surveillance, and follow-up is usually tailored to the individual after multidisciplinary review. We report a unique case of an ovarian lymphangioma coexisting with a haemorrhagic corpus luteal cyst, initially mistaken for a recurrent dermoid cyst after laparoscopic cystectomy. The patient, a woman in her 30s, presented several months after surgery with pelvic pain and prolonged menstrual bleeding. Ultrasound evaluation suggested a recurrent dermoid cyst. She subsequently underwent laparoscopic right salpingo-oophorectomy, and histopathological analysis confirmed the diagnosis. This case highlights the importance of maintaining a broad differential diagnosis when symptoms or lesions recur following complete dermoid excision. Histopathological evaluation is crucial to ensure diagnosis, while multidisciplinary discussion facilitates appropriate follow-up planning. Greater awareness of this rare entity may help clinicians avoid misdiagnosis and implement individualised postoperative surveillance.

## Introduction

Lymphangiomas are benign lymphatic vessel malformations that are most frequently seen in children, with intra-abdominal presentations being uncommon and ovarian involvement exceptionally rare [1]. Few well-documented similar ovarian cases have been reported, underscoring how unfamiliar this entity remains in gynaecological practice [2-4]. Preoperative diagnosis is challenging because the ultrasound findings usually show a multiloculated cystic mass with thin septations and mixed echogenicity, closely resembling more common adnexal lesions such as mature cystic teratoma, cystadenoma, or endometrioma [2]. Even with advanced imaging modalities, such as MRI, distinguishing ovarian lymphangioma from other cystic tumours is often difficult, leading to frequent misclassification. Therefore, histopathological examination remains the diagnostic gold standard.

Microscopically, ovarian lymphangiomas comprise thin-walled, endothelium-lined vascular channels containing serous or chylous fluid. Immunohistochemistry demonstrates positivity for endothelial markers such as CD31, CD34, and D2-40 (podoplanin). D2-40 (podoplanin) is considered the most specific immunohistochemical marker for lymphatic endothelium because it recognises a mucin-type transmembrane glycoprotein that is selectively expressed on lymphatic endothelial cells but absent on vascular (blood) endothelial cells [2,5]. This feature differentiates lymphangiomas from other cystic ovarian lesions, including dermoid cysts, which contain ectodermal derivatives such as skin, hair, and sebaceous tissue.

Management is primarily surgical, depending on patient age, fertility considerations, lesion size, and intraoperative findings. Options range from cystectomy to oophorectomy. The prognosis is generally favourable following complete excision. Because of the rarity of this tumour, no evidence-based postoperative surveillance guidelines exist. Therefore, follow-up is typically individualised through multidisciplinary discussion [6]. Recent case reports involving women from reproductive to postmenopausal ages support these findings and show that the condition can also affect one or both ovaries [2,4].

Within this context, our patient, a woman in her 30s, who initially underwent laparoscopic excision of a right ovarian dermoid cyst, later presented with pelvic pain and heavy, prolonged menstrual bleeding. Ultrasound suggested a recurrent dermoid cyst, but definitive histopathology following laparoscopic right salpingo-oophorectomy revealed a haemorrhagic corpus luteal cyst coexisting with an ovarian lymphangioma. However, clinical presentation is variable; some patients are asymptomatic, or the lesion is discovered incidentally during imaging or surgery for unrelated conditions.

This case highlights how rare lymphatic lesions can mimic more familiar adnexal pathology. It emphasises the importance of histological confirmation of the diagnosis in guiding management and informing individualised follow-up, particularly in the absence of standardised surveillance recommendations.

## Case presentation

Initial presentation and investigations

**Figure 1:**
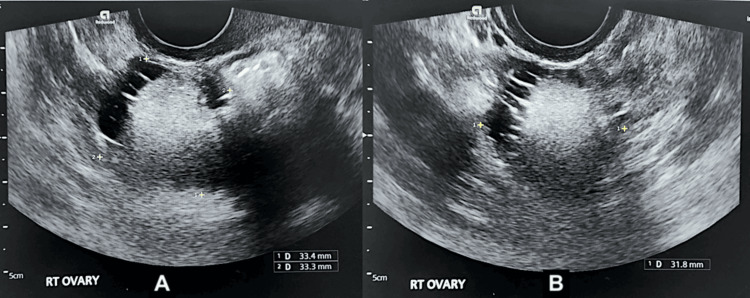
Transvaginal ultrasound of the right ovary demonstrating a dermoid cyst The right ovary (A, B) demonstrating a cyst measuring 33 × 33 × 32 mm. The lesion is predominantly solid with a small fluid-filled area and thin striations, with no internal vascularity on colour Doppler. Features are consistent with a dermoid cyst.

A woman in her 30s (Gravida 1, Para 1) presented with irregular menstrual cycles, menstrual-associated migraines, and intermittent lower abdominal pain. She had previously been assessed in a cancer-exclusion clinic, where a transvaginal ultrasound identified a right ovarian mass measuring 33 × 33 × 32 mm. The lesion appeared predominantly solid with a small fluid component and thin internal striations, with no vascularity on Doppler imaging. It was interpreted as a mature cystic teratoma (dermoid cyst) (Figure 1).

Her medical history included an overactive bladder managed with tolterodine. She was a smoker, reported no known drug allergies, and had no family history of malignancy. Tumour markers (CA-125, AFP, β-hCG, LDH, and CEA) were within normal limits, and preoperative blood tests were unremarkable.

First surgery and outcome

**Figure 2:**
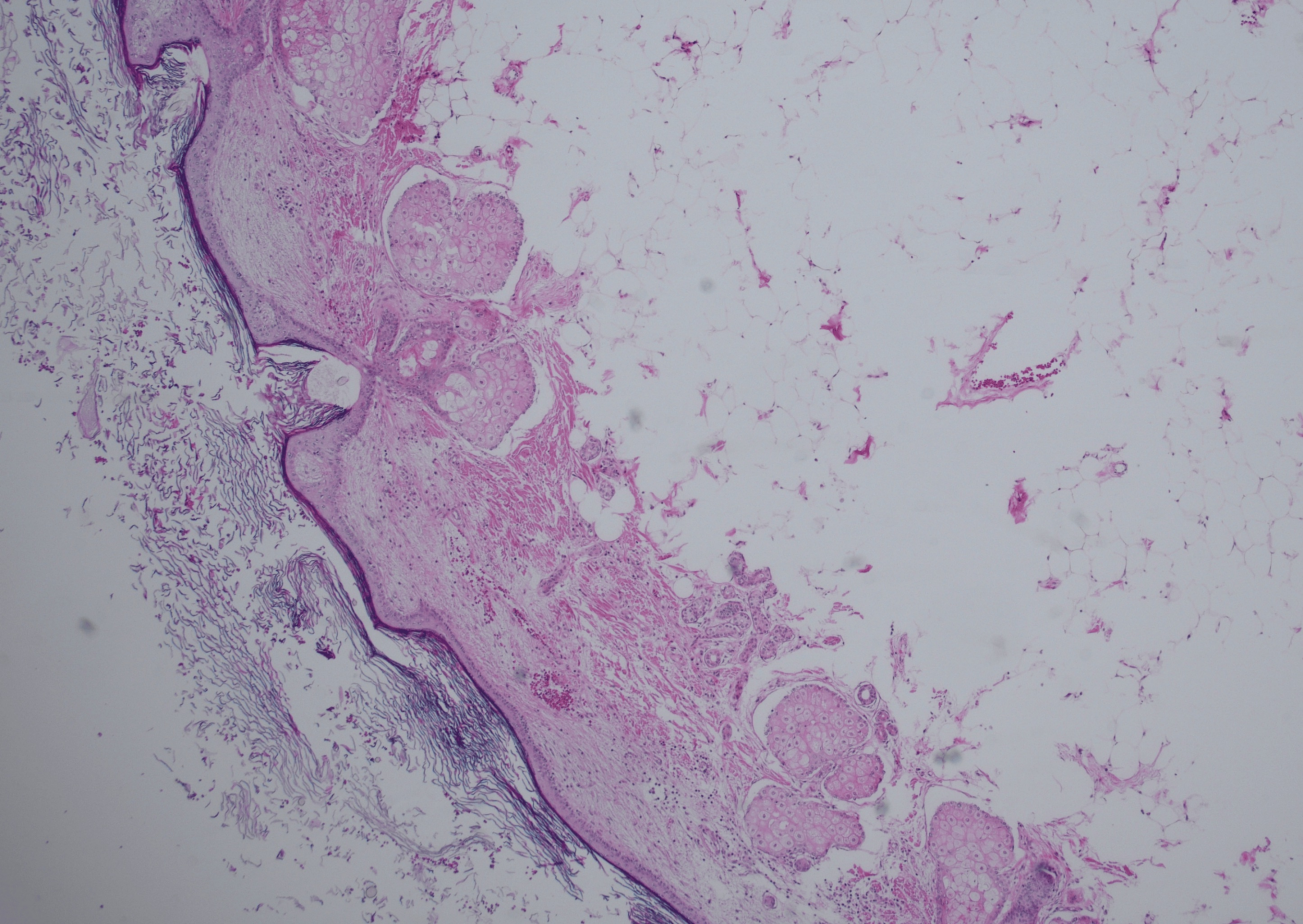
Histopathological evaluation of the right ovarian cyst demonstrates a mature cystic teratoma The right ovarian cyst comprises mature tissue elements, including skin appendages and adipose tissue, consistent with a mature cystic teratoma.

A laparoscopic right ovarian cystectomy was performed in late 2023. Intraoperatively, the cyst ruptured, releasing hair and sebaceous material, and the cyst wall was excised completely. The procedure and postoperative recovery were uneventful. Histopathological evaluation confirmed a mature cystic teratoma (Figure 2).

Recurrence and second surgery

**Figure 3:**
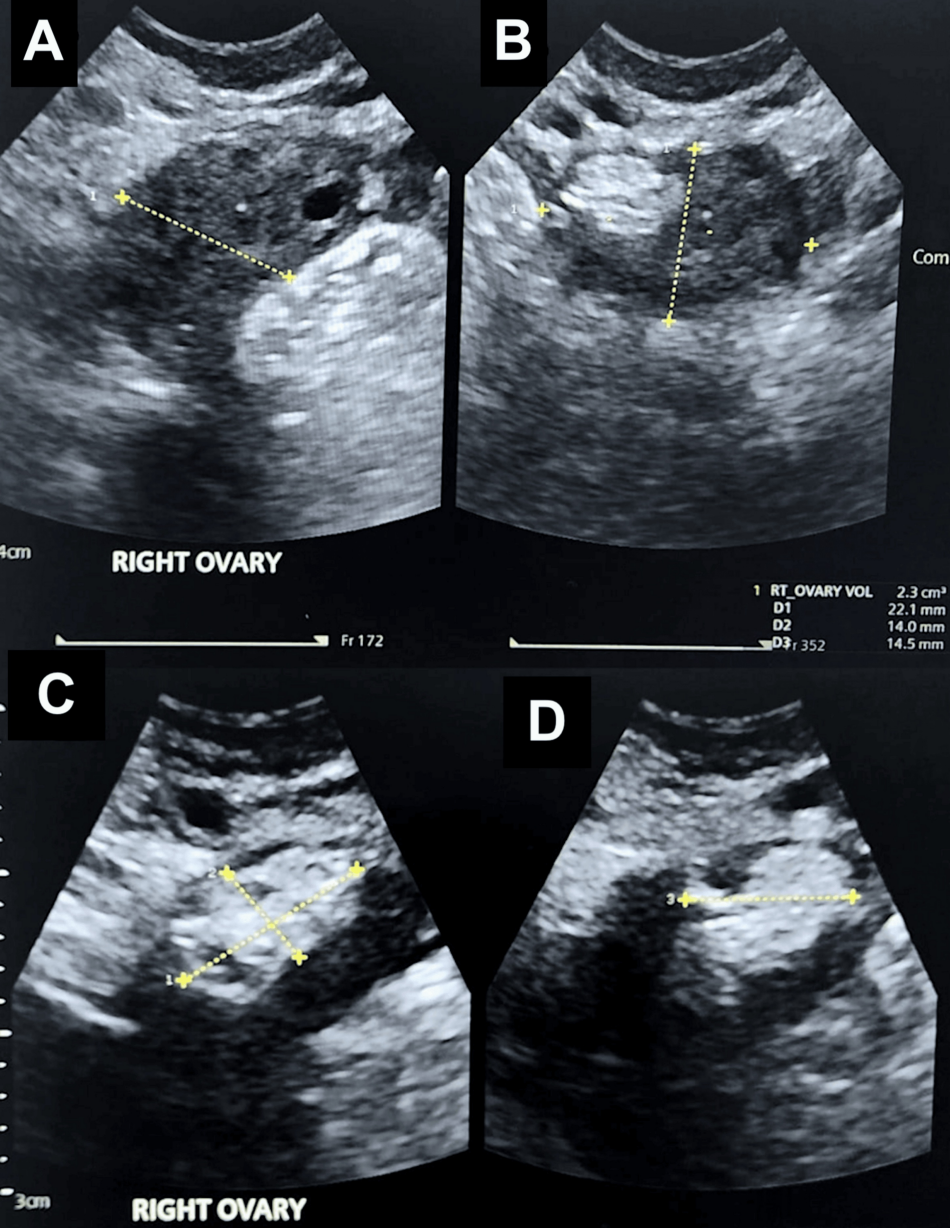
Transvaginal ultrasound of the right ovary demonstrating a suspected right ovarian dermoid cyst (A–B) Right ovary. (C–D) A hyperechoic, solid-appearing cyst containing multiple tiny cystic areas, measuring 12.9 x 6.9 x 10.5 mm, with no vascularity demonstrated on colour Doppler. The appearances are suggestive of a right ovarian dermoid cyst.

At a review about one year later, the patient reported pelvic pain and increasingly heavy, prolonged menstrual bleeding. Transvaginal ultrasound revealed a hyperechoic, solid-appearing cyst within the right ovary, containing multiple tiny cystic spaces and measuring 13 × 11 × 7 mm. No vascularity was demonstrated on Doppler imaging. The appearance suggested a possible recurrent dermoid cyst (Figure 3), and the tumour marker CA-125 remained within normal limits. She was treated with tranexamic acid for menorrhagia but declined hormonal therapy. After discussion of management options, including repeat cystectomy versus oophorectomy, she elected to undergo laparoscopic right salpingo-oophorectomy, which was planned preoperatively.

The operation, performed the following year, revealed a 50 × 40 mm cystic structure within the right ovary. Macroscopically, it resembled a dermoid cyst. The right ovary and fallopian tube were excised en bloc. The procedure was carried out as a routine salpingo-oophorectomy without complications, and postoperative recovery was uneventful.

Histopathology and follow-up

**Figure 4:**
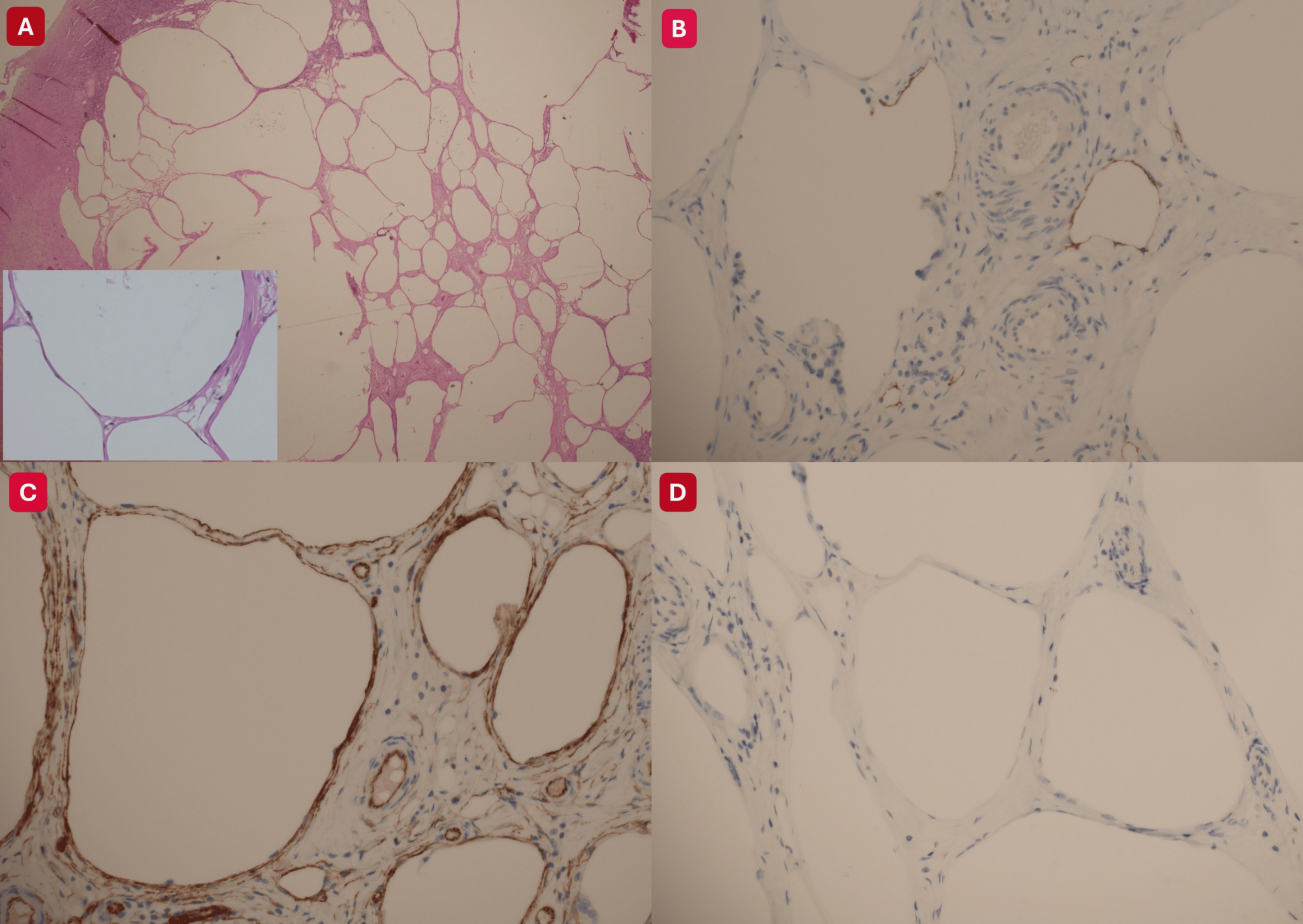
Histopathological evaluation of the right ovarian cysts reveals a benign lymphangioma The lymphangioma is composed of back-to-back thin-walled vascular channels lined by bland endothelium (A). The endothelial cells show flat nuclei without atypia (insert in A). The lining endothelial cells are focally positive for D2-40 (B), diffusely positive for CD34 (C), and negative for calretinin (D).

The macroscopic findings of the specimen consisted of an intact fallopian tube with fimbriae, measuring 47 × 6 mm, with a congested serosa surface. The right ovary measured 32 × 18 × 25 mm. On sectioning, the ovarian cut surface appeared cystic with focal haemorrhagic areas. Additionally, the microscopic examination revealed a haemorrhagic corpus luteal cyst and multiple back-to-back thin-walled vascular channels lined by non-atypical endothelium (CD34 positive, focally D2-40 positive, calretinin and WT1 negative), consistent with a benign lymphangioma. Benign follicular cysts were also present, and the fallopian tube was unremarkable. No dermoid tissue, atypia, or malignancy was identified (Figure 4).

The case was reviewed at a multidisciplinary team meeting. In view of the rarity of ovarian lymphangiomas and the lack of standardised follow-up protocols, annual pelvic ultrasound surveillance for two years was advised. At postoperative review, the patient remained clinically well, continued on tranexamic acid for heavy menstrual bleeding, and her pelvic ultrasound showed no abnormal findings.

## Discussion

Ovarian lymphangioma is among the rarest benign tumours in the ovary. Although lymphangiomas are typically congenital lesions of the head, neck and axilla, they may also arise in intra-abdominal organs such as the mesentery, retroperitoneum, and, very rarely, the ovary [2,7]. The exact cause of ovarian lymphangioma is not clearly understood, since lymphatic channels are normally scarce in ovarian tissue. However, several possible mechanisms have been proposed. The congenital theory suggests that during embryonic development, some lymphatic vessels become trapped within the developing ovary and later undergo cystic dilatation and proliferation due to their failure to connect with the main lymphatic system [8,9]. The acquired theory suggests that lymphangiomas can develop when existing lymphatic vessels become damaged or blocked due to inflammation, infection, trauma, or previous surgery. This disruption leads to local lymphatic stasis and stimulates endothelial cell proliferation, eventually forming cystic lesions [7,9]. Another mechanism, reactive in nature, was described, suggesting that chronic inflammation may trigger the overgrowth and dilation of preexisting lymphatic vessels within the ovarian stroma [2,5].

In our case, the lymphangioma was identified approximately one year after laparoscopic cystectomy on the same ovary, supporting a likely acquired or reactive pathogenesis, rather than a purely congenital anomaly. It is possible that surgical manipulation during the initial operation disrupted local lymphatic vessels or caused partial blockage, resulting in lymphatic stasis and gradual endothelial proliferation over time. Similar post-surgical mechanisms have been reported in cases of intra-abdominal lymphangiomas [7-9]. The coexistence of a haemorrhagic corpus luteal cyst appears incidental and unrelated to the lymphangiomatous process.

Histologically, these lesions consist of multiple thin-walled vascular spaces lined by flattened endothelial cells containing serous or chylous fluid. Immunohistochemical positivity for D2-40 (podoplanin) and endothelial markers such as CD31 and CD34 confirms their lymphatic origin and distinguishes them from other cystic ovarian tumours [3].

Clinical presentation is variable. Some patients experience abdominal or pelvic pain, menstrual irregularities, or pressure symptoms, while others remain asymptomatic and are diagnosed incidentally.

On imaging, ovarian lymphangiomas typically appear as multiloculated cystic masses with thin septations and variable echogenicity features that closely mimic dermoid cysts, cystadenomas, or endometriomas [2,4,9]. Even advanced imaging, such as MRI, cannot reliably differentiate lymphangioma from other complex cysts; therefore, histopathological assessment after excision remains the diagnostic gold standard [10].

Surgical excision is the mainstay of treatment, either by cystectomy or oophorectomy, depending on patient age, fertility wishes, and intraoperative findings [4,11]. Prognosis is excellent following complete removal, with recurrence or long-term complications rarely reported in the literature. Owing to the scarcity of cases, no evidence-based surveillance protocol exists. Most authors recommend ultrasound or MRI at 6- to 12-month intervals for one to two years, particularly when the lesion is large or excision margins are uncertain [5,9,12].

In our patient, annual pelvic ultrasound surveillance for two years was recommended following a multidisciplinary discussion, taking into account the benign nature of the tumour, the completion of oophorectomy, and the lack of established follow-up guidelines.

## Conclusions

Ovarian lymphangioma represents an exceptionally rare benign tumour that can closely mimic more common adnexal cystic lesions, posing significant challenges in preoperative diagnosis. This case illustrates how a lymphangioma can present after prior ovarian surgery and resemble a recurrent dermoid cyst, emphasising the importance of maintaining a broad differential diagnosis when evaluating complex ovarian lesions. Definitive diagnosis relies on histopathological and immunohistochemical confirmation, particularly with D2-40 as a specific marker of lymphatic endothelium. Complete surgical excision remains curative in most cases, and recurrence or long-term complications are exceedingly uncommon.

Without established surveillance protocols, multidisciplinary discussion plays a vital role in determining appropriate postoperative follow-up. Enhanced awareness of this uncommon pathology may help clinicians avoid diagnostic pitfalls and ensure optimal management in future cases.
